# Changes in coercive parenting and child externalizing behavior across COVID-19 and the moderating role of parent-child attachment relationship quality

**DOI:** 10.1371/journal.pone.0290089

**Published:** 2023-10-12

**Authors:** Sara I. Hogye, Nicole Lucassen, Katrien O. W. Helmerhorst, Paula Vrolijk, Renske Keizer

**Affiliations:** 1 Department of Public Administration and Sociology, Erasmus University Rotterdam, Rotterdam, The Netherlands; 2 Generation R Study Group, Erasmus MC University Medical Center Rotterdam, Rotterdam, The Netherlands; 3 Department of Child & Adolescent Psychiatry/Psychology, Erasmus MC University Medical Centre Rotterdam, Rotterdam, The Netherlands; 4 Department of Psychology, Education and Child Studies, Erasmus University Rotterdam, Rotterdam, The Netherlands; 5 Department of Pedagogy and Educational Sciences, Faculty of Behavioural and Social Sciences, University of Groningen, Groningen, The Netherlands; Tallinn University: Tallinna Ulikool, ESTONIA

## Abstract

Research indicates increases in coercive parenting towards children and increases in child externalizing behavior during COVID-19 as compared to the pre-pandemic period. In this preregistered study, we extended previous knowledge by investigating to what extent, and under what conditions, changes in coercive parenting and child externalizing behavior are interrelated. Ninety-five mothers and fathers of children (of age 3 prior to the pandemic) reported on coercive parenting and child externalizing behavior before and during the pandemic, and trained assistants observed the quality of mother-child and father-child attachment relationship prior to the pandemic. We employed latent change score modeling to test the extent to which changes in maternal and paternal coercive parenting and changes in child externalizing behavior across the pre-pandemic period and the onset of the first COVID-19 lockdown are interrelated. Moreover, we tested whether these linkages are moderated by changes in the other parent’s coercive parenting and the quality of parent-child attachment relationship. Specifically, we tested the moderation by mother-child (father-child) attachment relationship quality in the relation between changes in mothers’ (fathers’) coercive parenting and changes in child externalizing behavior. We found that changes in mothers’, but not fathers’ coercive parenting were positively associated with changes in child externalizing behavior. We found no moderation by changes in the other parent’s parenting or by parent-child attachment relationship quality. Our findings provide support for the transactional processes underlying mothers’ and children’s behavior in the context of non-normative stressful conditions. We recommend incorporating evidence-based (parenting) support for mothers, fathers, and young children in prevention strategies and recovery tools employed during and after future lockdowns and non-normative stressful events.

## Introduction

The COVID-19 pandemic and regulations to contain the virus (e.g., lockdowns) have been related to a variety of changes in the family environment. Specifically, parents reported using less effective parenting practices, including coercive parenting toward three- and four-year-old children, during COVID-19 as compared to the pre-pandemic period [1]. These findings have also been reported in a study using the same data as the present study [2]. In addition, as compared to the pre-pandemic period, on average children experienced higher levels of externalizing behavior during COVID-19 [3–9], although some studies did not confirm changes in child externalizing behavior [10] or deviations from normative sample (i.e., pre-pandemic) scores [11]. In the current study, using longitudinal data from the pre-pandemic period to the onset of the first COVID-19 lockdown, we extended prior research that assessed relations between *changes* in coercive parenting and *changes* in child externalizing behavior in three notable ways. First, previous studies on linkages between changes in coercive parenting and changes in children’s behavior often overlooked the fact that subsystems within the family (e.g., mother-child dyad, father-child dyad) are interrelated and influence one another [12]. Studies frequently focused solely on the role of mothers’ or the ‘primary caregivers’ coercive parenting [7], or used an aggregated measure of mothers’ and fathers’ coercive parenting [13, 14], even though associations between coercive parenting and child outcomes may differ depending on which parent shows coercive parenting [15]. In the current study, we examined mothers’ and fathers’ coercive parenting simultaneously in relation to changes in child externalizing behavior (RQ1). Second, as the association between one parent’s coercive behavior and their child’s externalizing behavior likely differs depending on the extent to which the other parent also shows coercive behavior, we considered the interaction between changes in mother’s and fathers’ coercive parenting in relation to changes in children’s externalizing behavior (RQ2).

Third, we investigated whether protective factors within the parent-child relationship moderate the relations between changes in coercive parenting and changes in child externalizing behavior. As not all parents and children are influenced to the same extent by environmental stressors that stem from outside of the family [16], it is essential to gain a better understanding of protective factors that are manifested in the parent-child dyad. The quality of the parent-child attachment relationship may be a potential moderator in the interrelated changes between coercive parenting and child externalizing behavior given that a secure parent-child attachment relationship is beneficial for child developmental outcomes [17], and it may provide a foundation for children to rely on in sudden stressful contexts. Therefore, we examined to what extent the quality of mother-child and father-child attachment relationships, assessed during the pre-pandemic period, may moderate the relation between changes in maternal and paternal coercive parenting and changes in child externalizing behavior (RQ3).

### Interrelated changes in coercive parenting and child behavior

Coercive parenting falls under the umbrella term of negative parenting and characterizes harsh and coercive acts by the caregiver towards the child, such as shouting, guilt inducing, and smacking [18, 19]. Theoretical frameworks on social learning [20] and coercive family processes [21, 22] outline the transactional processes underlying the bidirectional relation between coercive parenting and child externalizing behavior. According to social learning theory [20], through modeling of parents’ inappropriate behavior or behavior that is ineffective for coping, children learn to use aggressive, hostile, or aversive behavior to solve problems, conflicts, or disagreements. Both the parent and child may mutually reinforce each other’s coercive behavior in reciprocal interactions. For instance, in a particular situation, a parent’s coercive reaction to a child’s misbehavior may, in turn, exacerbate or maintain the child’s misbehavior [23]. Such negative reinforcements could fuel future coercive parent-child interactions, which in turn, may create, shape, and maintain coercive family processes over time [21, 22, 24]. It is likely that if family members provide negative reinforcements, which are more powerful than positive reinforcements in maintaining the coercive behavior, coercive interactions may escalate in intensity [23]. Coercive parent-child interactions may also influence interactions with other family members, which, through positive or negative reinforcements, may also shape coercive family processes over time as well as children’s social interactions outside of the family environment [23].

Accordingly, there is robust evidence of concurrent, longitudinal, and cross-lagged relations between coercive parenting and child externalizing behavior [25]. Results from the meta-analysis by Yan et al. [26] underscore the similarity of parent and child-driven effects in the relations between negative parenting practices, including coercive parenting, and child externalizing behavior. Moreover, studies conducted in the context of COVID-19 suggest that changes in coercive parenting and changes in child externalizing behavior over time may be interrelated [7, 14]. For example, Fosco et al. [14] found that increases in harsh parenting from before to during the pandemic were associated with more child externalizing behavior during the pandemic, after controlling for pre-pandemic levels of child externalizing behavior, parent distress, and financial strain. Khoury et al. [7] found that maternal hostility/coerciveness (i.e., losing temper with child, threatening child) during the pandemic was related to greater increases in child externalizing behavior from before to during COVID-19, after controlling for pre-pandemic levels of child mental health, maternal mental health, and stress. Overall, previous research shows that increases in coercive parenting from before to during the pandemic are related to increases in child externalizing behavior.

### Mothers’ and fathers’ coercive parenting and child externalizing behavior

Family systems theory proposes that subsystems within the family are interconnected, and continuously and reciprocally influence one another [12]. As such, in families with two caregivers, it is essential to study the association between parenting and child behavior across both caregivers. Despite moderate to strong correlations between mothers’ and fathers’ coercive parenting [27–30], when maternal and paternal parenting was examined simultaneously in relation to child problem behavior, mixed findings arose across mothers and fathers. Specifically, some studies highlighted stronger associations between parenting and child externalizing behavior for mothers than fathers [18, 29, 31], while other studies highlighted stronger associations for fathers than mothers [30, 32]. However, studies have not always statistically tested differences in the strength of parenting-child behavior association across mothers and fathers, which may have led to erroneous conclusions [33]. Meta-analyses that contrasted the associations between parenting and child behavior across mothers and fathers revealed mixed support for differential associations across mothers and fathers. Contrary to the meta-analyses by Rothbaum and Weisz [34] and Hoeve et al. [35] where the authors identified differences in the strengths of associations between parenting and child and adolescent externalizing behavior across mothers and fathers, findings from three subsequent meta-analyses [25, 36, 37] revealed no differences in the associations across mothers and fathers. Therefore, in line with the family systems perspective and the growing empirical evidence indicating no differences in parenting-child behavior associations across mothers and fathers, we expect that changes in both mothers’ and fathers’ coercive parenting are independently and similarly related to changes in child externalizing behavior.

Complementary to studying additive associations, we applied insights from the family systems theory by considering the interaction between mothers’ and fathers’ coercive parenting in relation to child externalizing behavior. Interactions may represent three patterns [38]: One parent’s coercive parenting may *strengthen* (i.e., amplify), *interfere* with (i.e., hinder), or *buffer* (i.e., weaken) the association between the other parent’s coercive parenting and child externalizing behavior. A strengthening pattern would imply that one parent’s increases in coercive parenting would be more strongly related to increases in child externalizing behavior when the other parent also increases in coercive parenting. An interfering pattern would imply that the association between increases in one parent’s coercive parenting and increases in child externalizing behavior would be hindered (the association would be weaker) when the other parent also increases in coercive parenting (i.e., increases in the other parent’s coercive parenting interferes with the association). A buffering pattern would imply that the relation between increases in one parent’s coercive parenting and increases in child externalizing behavior would be weaker in the condition that the other parent decreases (or increases less) in coercive parenting.

Empirical support for the potential interplay between mothers’ and fathers’ coercive parenting in relation to child externalizing behavior comes from different strands of research on negative parenting. Braza et al. [39] found that the interaction between an authoritarian (high firm control and low warmth) maternal style and a permissive (high warmth and low control) paternal style was positively related to children’s physical and indirect aggression, suggesting a strengthening pattern of interaction. The authors also reported a strengthening pattern of interaction between permissive maternal and permissive paternal style in relation to girls’, but not boys’, physical aggression [39]. Similarly, Mendez et al. [33] found that mothers’ harsh parenting strengthened the relation between fathers’ corporal punishment and child externalizing behavior. In addition to a strengthening pattern, Braza et al. [39] also found an interfering pattern of interaction between authoritarian maternal and authoritarian paternal styles, which was negatively related to child externalizing behavior and indirect aggression. Roskam et al. [30] instead found support for a buffering role of low parental control in the association between control by the other parent and child externalizing behavior. Thus, on the one hand increases in both parents’ coercive parenting may reflect either a strengthening or interfering pattern of interplay in relation to child externalizing behavior, on the other hand, decreases in one parent’s coercive parenting may instead reflect a buffering pattern. Still, Lee et al. [29] did not find an interaction effect between maternal and paternal spanking on child aggression at age three or five years. Given the inconsistency in previous findings on the potential pattern of interplay between mothers’ and fathers’ parenting, we explore to what extent changes in one parent’s coercive parenting interact with changes in the other parents’ coercive parenting in relation to changes in child externalizing behavior.

### Parent-child attachment as a moderator

The quality of the parent-child attachment relationship is typically defined as the emotional bond that a child forms with their caregiver based on the extent to which the child views their caregiver as a secure base from which they can independently explore, a safe haven to come back to when they are distressed, and the quality of sensitive responsiveness they receive from their caregivers in times of distress [17, 40]. The quality of the parent-child attachment relationship plays a crucial role in children’s emotional and behavioral development [17]. In early childhood, children form internal working models of attachment with several attachment figures. Internal working models include internalized representations of attachment relationships a child has and can forecast to what extent one’s social environment is dependable and trustworthy across a range of situations [41]. Based on attachment theory, we argue that in contexts characterized by higher levels of stress that originates outside of the family, children with a higher quality parent-child attachment relationship continue to rely on internal working models that promote their view of their social environment as trustworthy and dependable. Specifically, in sudden relatively stressful contexts, such as the COVID-19 pandemic and related regulations, children with a higher quality parent-child attachment relationship who experience increases in coercive parenting (such as during a lockdown) may continue perceiving their parents and social environment as trustworthy and dependable, and thus may be less susceptible to changes in the family environment. As such, among children with a higher quality parent-child attachment relationship, the interrelated association between changes in coercive parenting and child externalizing behavior may be weaker than among children with a lower quality attachment relationship. In a similar vein, we expect that a lower quality parent-child attachment relationship may amplify the association between changes in coercive parenting and child externalizing behavior.

Empirical evidence suggests that the quality of parent-child attachment relationships may provide a context in which the coercive cycle between coercive parenting and child externalizing behavior operates. For example, Cyr et al. [42] found that only among children with an insecure attachment, and not a secure attachment, maternal criticism (e.g., making statements about negative attributes of child) observed at four-and-a-half years was associated with child aggression in grade one. Similarly, Ward et al. [43] found that among children with an insecure mother-child attachment relationship, and not with a secure mother-child attachment relationship, maternal spanking at child age one was related to child externalizing behavior at age three. However, mother-child attachment relationship quality did not moderate the association at later ages.

The few studies that assessed the moderating role of father-child attachment relationship in addition to the moderating role of mother-child attachment relationships found similar effects for mother-child and father-child attachment relationships [27, 28, 44]. In their longitudinal prospective study, Kochanska et al. [27] found that parental power assertion (incl. salient pressure, forceful or harsh insistence, criticism), via child resentful opposition, was related to child antisocial and disruptive behavior among children who were insecurely, but not among those who were securely attached to their parent. Similarly, across multiple studies, Kochanska et al. [28] found the pattern that only among children with an insecure parent-child attachment relationship, child anger proneness or difficult temperament was associated with power-assertive parenting and heavy-handed control, which were associated with child antisocial behavior. Overall, empirical evidence supports the moderating role of the quality of both mother-child and father-child attachment relationships in the associations between coercive parenting and child externalizing behavior.

### The current study

In the current preregistered study, we used a multi-informant, prospective, longitudinal study design to assess to what extent changes in coercive parenting are related to changes in child externalizing behavior in the context of COVID-19. First, we hypothesized that changes in maternal and paternal coercive parenting will be independently and positively related to changes in child externalizing behavior (*H1*). We explored to what extent the association between changes in maternal coercive parenting and changes in child externalizing behavior differs from the association between changes in paternal parenting and changes in child externalizing behavior. Second, we explored to what extent changes in one parent’s coercive parenting interact with changes in coercive parenting by the other parent in relation to changes in child externalizing behavior (exploratory *H2*). Third, we hypothesized that the more secure the attachment relationship is, the weaker the association between changes in coercive parenting and changes in child externalizing behavior (*H3*).

Our study contributes to the literature in multiple ways. Previous studies conducted in the initial phase of the COVID-19 pandemic were often limited to a cross-sectional study design [11], which did not allow for tests of changes over time; or relied on retrospective assessments of parent and child behavior [4, 13], which may have entailed recall bias. In order to reliably test the interrelated changes in coercive parenting and child externalizing behavior within the same families over time, in our study, we included prospective reports on parent and child behavior prior to the pandemic and the onset of the first lockdown in the Netherlands. In the present study, we employed latent change score modeling, a type of longitudinal structural equation modeling that accounts for measurement error, to model changes in parent and child behavior over time [45].

In addition, previous research that studied solely the role of mothers’ or a combination of maternal and paternal coercive parenting in relation to child externalizing behavior [7, 14] may have overlooked the independent and additive associations of maternal and paternal coercive parenting in relation to child behavior. In line with the family systems theory, in our study we scrutinized the role of both mothers’ and fathers’ coercive parenting. Therefore, we investigated the joint as well as the interactive associations of changes in both mothers’ and fathers’ coercive parenting in relation to changes in child externalizing behavior, and we examined to what extent the quality of mother-child and father-child attachment relationships could moderate the interrelated changes in coercive parenting and child externalizing behavior. In order to do so, we used multi-actor data: besides including both mothers’ and fathers’ reports on coercive parenting and child externalizing behavior, independent observers assessed the quality of mother-child and father-child attachment relationships.

## Methods

### Study design and participants

The present study is embedded in an interdisciplinary research project that investigates the role of mothers and fathers, from the same family, in children’s development. Student assistants recruited families at playgrounds, national festivities, swimming pools libraries, and general outdoors in Rotterdam, in the Netherlands. Families were eligible if (a) their child was three years old at the time of recruitment, (b) both mothers and fathers lived with one or more children in the same household, and (c) both parents had a native Dutch background (both parents of the participating parents were born in the Netherlands).

Data for the first wave (T1) were collected between May 2018 and January 2020 (*N* = 104), prior to the detection of the first case of COVID-19 in the country (February 27, 2020) and the onset of the first lockdown in the Netherlands (March 12, 2020). Data for the second wave (T2; 96% response rate, *N* = 100) were collected between April 15, 2020 and May 11, 2020 during the first lockdown in the Netherlands. The difference in time between measurements at T1 and T2 ranges between 3 to 23 months, median (interquartile range) = 16 (11–19.5). From the 104 families, we excluded four families in which the parents had separated in the period between the two study waves, and we excluded five families who did not participate at T2. From the remaining 95 families, 1% of mothers and 6% of fathers had missings on coercive parenting on scale level at T2, and 2% of mother-reported and 6% of father-reported child externalizing behavior were missing on scale level.

### Procedure

At T1, two trained research assistants visited the participating families at home. During these visits, fathers and mothers were each observed separately with their child (by different independent observers), and each parent-child session lasted a total of two hours. The order of the parent-child sessions (mother or father first) was counterbalanced across families. During the first 30 minutes of each parent–child session, the parent was asked to fill in an online questionnaire using a tablet. The research assistants did not interfere with the parent while they were filling in the questionnaire. Children were instructed to play by themselves while their parent filled in the questionnaire. After filling in the questionnaire, the parent and their three-year-old were asked to perform several play tasks together (free play without toys, building blocks, etch-a-sketch, horse-riding on parent’s back, sock wrestling, and free play with toys). One research assistant observed parent-child attachment during the entire parent-child session, while the other assistant was the instructor for the play tasks and the one who made the video recordings. Between the father-child session and mother-child session, assistants switched tasks.

At T2, due to the lockdown measures in the Netherlands, families were not visited at home, but instead, all participating mothers and fathers were asked to complete questionnaires digitally. The Ethics Committee of the Department of Public Administration and Sociology, Erasmus University Rotterdam approved both waves of data collection. All parents provided written informed consent for their participation and for their children’s participation.

### Measures

#### Coercive parenting

Mothers and fathers self-reported on the coercive parenting subscale of the Parenting and Family Adjustment Scales (PAFAS) [19] at both time points. The PAFAS was designed as a brief inventory to study changes in parenting practices and parent and family adjustment in the evaluation of public health and parenting interventions [19]. At T1, parents were asked to report on a 4-point scale ranging from 0 = “not true of me at all” to 3 = “true of me very much” on the extent to which the five items of the coercive subscale applied to them in the past four weeks. At T2, parents were asked to report on the items at the *current time period of the lockdown*. An example item is “I shout or get angry with my child when they misbehave”. For the main analyses, we used items from the coercive parenting subscale as observed indicators underlying the latent construct coercive parenting, which we created separately for mothers and fathers (see created latent constructs in the Supplementary Information under Figure S1 in the S1 File). Based on tests of longitudinal measurement invariance, which we explain in detail in the section Analytic Strategy, we excluded one original item (“I spank [smack] my child when they misbehave”) from the latent construct for both mothers and fathers. Sanders et al. [19] reported that the coercive parenting subscale had good internal consistency and satisfactory construct and predictive validity. Cronbach’s alphas for maternal and paternal coercive parenting without the excluded item at T1 and T2 ranged between .58 and .61. The low Cronbach’s alpha values are comparable with Morawska et al.’s [46] study that included mainly mothers. Furthermore, as Cronbach’s alpha is, in part, a function of the number of items, it is not clear that the usual rules of thumb apply for scales with only a select number of items, which tap into different types of coercive parenting behavior, such as shouting and guilt inducing [47]. To report descriptives, separately for mothers and fathers, we summed up the items included in the main analyses to compute a composite score for overall coercive parenting, ranging between 0–12, with higher scores reflecting higher levels of coercive parenting.

#### Child externalizing behavior

Mothers and fathers reported on child externalizing behavior using the Strengths and Difficulties Questionnaire (SDQ) [48, 49]. The SDQ is a brief behavioral screening questionnaire that includes 25 items with both negative and positive attributes of child behavior. At T1, parents indicated to what extent the attributes applied to their child in the past six months on a 3-point Likert scale ranging from 0 = “not true” to 2 = “certainly true”. At T2, parents indicated to what extent the attributes applied to their child at the *current time period of the lockdown*. Two subscales of the SDQ assess externalizing behavior, consisting of five items each: Hyperactive/Inattention (e.g., “Constantly fidgeting or squirming”, “Easily distracted, concentration wanders”) and Conduct problems (e.g., “Often fights with other children or bullies them”). We recoded reversed items. To achieve longitudinal measurement invariance, we excluded two items in the Conduct problems scale, as the items were phrased differently at T1 versus T2. The phrasing of item “often argumentative with adults” in the SDQ for 2–4 years-olds was adapted to “often lies or cheats” in the SDQ for 4-10-years-olds. The phrasing of the item “can be spiteful to others” in the SDQ for 2-4-years-olds was adapted to “steals from home, school, or elsewhere” in the SDQ for 4-10-years-olds.

For the main analyses, in line with recommendations [50], we created four parcels based on mean scores for the Conduct problems scale (separately for mothers and fathers) and Hyperactive/Inattention scale (separately for mothers and fathers) in order to reduce the number of parameters in the latent change score models. Using parcels instead of individual items as indicators for the underlying latent construct (i.e., externalizing behavior) allows us to have more parsimonious models [50]. We allowed all four parcels to load onto one latent factor (see created latent constructs in the Supplementary Information under Figure S2 in the S1 File). Goodman [48] reported satisfactory internal reliability, interrater reliability, and test-retest reliability of the SDQ in a sample of British children. More specific to children’s age range in our sample, satisfactory internal consistency of the externalizing behavior scale of the SDQ has been reported across multiple community samples of children aged between two to five years [51, 52]. In our sample, Cronbach’s alphas for the mother-reported and father-reported externalizing scales ranged between .75 and .79 across T1 and T2. To report descriptives, we created sum scores based on items from the Conduct problems scale and the Hyperactivity/Inattention scale separately for mothers and fathers, and we subsequently averaged the sum scores across mothers and fathers into one externalizing scale ranging between 0–16, with higher scores reflecting more externalizing behavior.

#### Quality of the parent-child attachment relationship

The quality of the mother-child and father-child attachment security (secure base behavior) was observed using the Attachment Q-Sort (AQS) [53] by thoroughly trained observers during the home visit at T1. During the home visit, the observers rated 90 items, including descriptions of a range of infant secure base behaviors, on a scale from 1 (“least descriptive of the child”) to 9 (“most descriptive of the child”). Example items of the child’s secure base behavior are “Child puts his arms around parent or puts his hand on parent’s shoulder when he/she picks him up”, “If held in parent’s arms, child stops crying and quickly recovers after being frightened or upset”. After the observations, the researchers sorted the 90 items into 9 piles, each containing 10 items. Subsequently, the child’s secure base behavior score was calculated by correlating the child’s score on all 90 items with the criterion sort of a prototypical securely attached child, which was established by a group of experts [53]. The child’s secure base behavior score can range from -1 (insecure attachment) to 1 (secure attachment).

The AQS has satisfactory convergent, discriminant, and predictive validity [54, 55]. An expert on the AQS trained K. O. W. H. and L. S., who subsequently trained all observers in the present research. Before scoring the AQS independently, all observers had to reach 80% agreement for three observations with K. O. W. H. and L. S. Inter-observer reliability was computed on 35 out of the total 208 parent-child observations during data collection, in which two trained observers assessed the parent-infant attachment relationships. Agreement between observers ranged between *r =* .71 and *r =* .99 and was *r =* .86 on average.

#### Covariates

Based on theoretical and empirical work [25, 52], we considered the following parent-reported variables as potential covariates in our study: child sex as reported at T1, and child age (in months) as reported at T2. We considered child age at T2 as a potential covariate given that there is more variability in child age at T2 as opposed to T1 (please see Table 1 for descriptive statistics). Moreover, the current study deviates in one point from the preregistration: In line with a reviewer’s suggestion, in sensitivity analyses, we additionally tested the time between measurement occasions as a covariate in our study.

**Table 1:** Descriptive statistics of study variables.

	Time 1				Time 2			
	*Mean*	*SD*	*Range* ^a^	*N*	*Mean*	*SD*	*Range* ^a^	*N*
Child age (in months)	41.27	3.89	36.00–47.00	95	56.08	7.12	41.00–69.00	95
Maternal coercive parenting	2.81	1.43	0.00–7.00	95	3.00	1.53	0.00–8.00	94
Paternal coercive parenting	2.50	1.51	0.00–7.00	95	2.87	1.66	0.00–8.00	89
Child externalizing behavior	5.40	2.70	0.50–15.50	95	5.581	3.10	0.00–15.50	93
Mother-child attachment relationship	0.40	0.23	-0.36–0.77	95	-	-	-	-
Father-child attachment relationship	0.45	0.22	-0.38–0.83	95	-	-	-	-

Note.

^a^ Observed range.

### Analytic strategy

We preregistered the present study on the Open Science Framework (https://doi.org/10.17605/OSF.IO/Y95AT). In line with a reviewer’s suggestions, we deviated from the preregistered analytic strategy (observed change scores), and we have tested the preregistered hypotheses using a latent change score framework. Latent change score modeling allows to study (1) the level of change in parent and child behavior across pre-pandemic (initial) levels and pandemic levels, and to (2) disentangle differences in changes between people (i.e., between-person changes) from changes that occur within individuals (i.e., within-person changes) [45].

We conducted our analyses in MPlus Version 8 [56], using Robust Maximum Likelihood (MLR) estimation. Little’s missing completely at random (MCAR) test [57] revealed that there is no evidence that the pattern of missingness in our study is not MCAR (χ^2^ = 37.170, *df* = 27, *p* = .09). We handled data that was missing on maternal coercive parenting (1%) and paternal coercive parenting (6%) at T2, and on mothers’ (2%) and fathers’ (6%) reports on child externalizing behavior at T2 using Full Information Maximum Likelihood (FIML).

Prior to conducting the main analyses, we conducted tests of full factorial measurement invariance across (1) measurement occasions (i.e., longitudinal measurement invariance) for maternal coercive parenting, paternal coercive parenting, and child externalizing behavior, and (2) reporter for child externalizing behavior. Specifically, we tested various levels of measurement invariance [58]. First, we tested configural variance (Model A), meaning that we examined whether the construct is theoretically operationalized in similar ways at T1 and T2, and in similar ways for mothers and fathers. In Model A, we did not impose constraints on any parameters. Second, we tested metric invariance (Model B), which implies that we examined whether the same meaning is attributed to the latent construct across T1 and T2, and across mothers and fathers. In Model B, we constrained the factor loadings to be equal across T1 and T2, and equal across mothers and fathers. Third, we tested scalar invariance (Model C), which implies that we examined whether the meaning of the construct including the levels of underlying items are equal across T1 and T2, and equal across mothers and fathers. In Model C, we additionally constrained the item intercepts to be equal across T1 and T2, and equal across mothers and fathers. Fourth, we tested strict invariance (Model D), which implies that we examined whether the latent construct is measured identically at T1 and T2, and identically for mothers and fathers. In Model D, we additionally constrained residual variances to be equal across T1 and T2, and equal across mothers and fathers.

If imposing invariance constraints resulted in a significant Satorra-Bentler Chi-square difference value (*p* < .05), and additionally in Δ_CFI_ ≥ -.005 supplemented by Δ_RMSEA_ ≥ .010 (item loadings) or Δ_SRMR_ ≥ .025 (item loadings) or Δ_RMSEA_ ≥ .010 (item intercepts), the respective type of constraints were not tenable [59]. If the more constrained model fit significantly worse, we made plausible adjustments to improve the fit of the measurement model based on model identification indices. We allowed the residual variances of parallel items (coercive parenting, child externalizing behavior) across measurement occasions to correlate [60].

To test our hypotheses, we conducted four multivariate latent change score models that included any adjustments made to the measurement models from the measurement invariance tests. The latent change score models allow us to examine group-level initial (T1) levels (i.e., intercepts) and changes across T1 and T2, as well as individual differences in intercepts and change scores. Considering the low sample size and limited power, we decided not to include covariates in the main analyses, given that neither child sex nor child age correlated with both maternal and paternal coercive parenting, and child externalizing behavior at T1 and T2. In sensitivity analyses, we tested time difference across measurement occasions as an additional control variable in Model 1.

In Model 1, we tested Hypothesis 1 on changes in maternal and paternal coercive parenting being positively and independently related to changes in child externalizing behavior. To do so, in Model 1, we examined the covariance (i.e., correlation) between changes in maternal coercive parenting, changes in paternal coercive parenting, and changes in child externalizing behavior, while statistically accounting for (a) the covariance between initial (T1) levels of coercive parenting and child externalizing behavior and (b) longitudinal coupling (i.e., regression) between initial levels of and changes in coercive parenting and child externalizing behavior. Using a Wald test of parameter constraints, we tested whether the association between changes in coercive parenting and changes in child externalizing behavior significantly differed between fathers and mothers.

In Model 2, we examined whether the relation between one’s parent’s coercive parenting with changes in child externalizing behavior was moderated by changes in the other parent’s coercive parenting (Hypothesis 2). To do so, we tested a latent variable interaction in the multivariate latent change score model using the XWITH command in Mplus. In order to test the interaction, instead of correlating change scores, we regressed change in child externalizing behavior on changes in maternal and paternal coercive parenting and the product term of the two.

We extended Model 1 into Model 3 and Model 4, where we examined moderation by the quality of parent-child attachment relationship in the relation between changes in parental coercive parenting and changes in child externalizing behavior (Hypothesis 3) separately for mothers (in Model 3) and fathers (Model 4) in a similar way as previously explained (XWITH in Mplus).

## Results

### Descriptive results

Descriptive statistics of child and parent characteristics can be viewed in Table 1. At both measurement occasions, the level of coercive parenting reported by mothers was not significantly different from that of fathers (T1: *t*(188) = 1.48, *p* = .140; T2: *t*(181) = .57, *p* = .568). The average observer-rated mother-child and father-child attachment relationship security scores at T1 did not differ significantly from each other (*t*(188) = -1.76, *p* = .080) and were comparable to the meta-analysis by Cadman et al. (2018) [54], indicating that in our sample, on average, children had a relatively secure parent-child attachment relationship quality. We reported correlations among the study variables at T1 and T2 in Table 2. Mother and father coercive parenting were positively correlated at T1 (*r* = .23, *p* = .024) and T2 (*r* = .34, *p* = .001). Child externalizing behavior was positively correlated with maternal coercive parenting (*r* = .25, *p* = .018) and paternal coercive parenting (*r* = .32, *p* = .002) at T2. Mother-child and father-child attachment relationship were positively correlated (*r* = .47, *p* < .001). Moreover, girls were more securely attached to their mothers than were boys (*r* = .28, *p* = .005).

**Table 2:** Correlations among study variables at time 1 (N = 95) and time 2 (N range between 89–95).

	1.	2.	3.	4.	5.	6.	7.
1. Maternal coercive parenting	-	.34**	.25*	-.09	-.11	.16	.17
2. Paternal coercive parenting	.23**	-	.32**	.15	.04	.13	.13
3. Child externalizing behavior	.12	.11	-	-.26	-.11	-.25*	-.16
4. Mother-child attachment relationship	-.09	.08	-.16	-	-	-.05	-
5. Father-child attachment relationship	-.03	.11	-.18	.47***	-	-.13	-
6. Child age^a^	.12	.16	-.31**	-.05	-.13	-	-
7. Child sex^b^	.15	.03	-.16	.28**	.16	.09	-

*Note*. Bottom half of the table features correlations for Time 1, and top half features correlations for Time 2.

All correlations represent Pearson correlations, except those with child sex (boys coded as 0 and girls coded as 1) represent point biserial correlations.

* *p* < .05,

** *p* < .01,

*** *p* < .001.

^a^ Child age assessed at T2.

^b^ Child sex assessed at T1.

### Measurement invariance

We established longitudinal measurement invariance for maternal and paternal coercive parenting and child externalizing behavior, and measurement invariance across reporter for child externalizing behavior. However, we made small adjustments to the measurement models, which we explain in detail in the Supplementary Material, under Measurement Invariance Tests (see also Supplementary Tables S1 and S2 in the S1 File for model fit indices, model comparisons, and specific alternations to the models).

### Multivariate latent change score models

#### Interrelated changes in coercive parenting and child externalizing behavior

**Fig 1:**
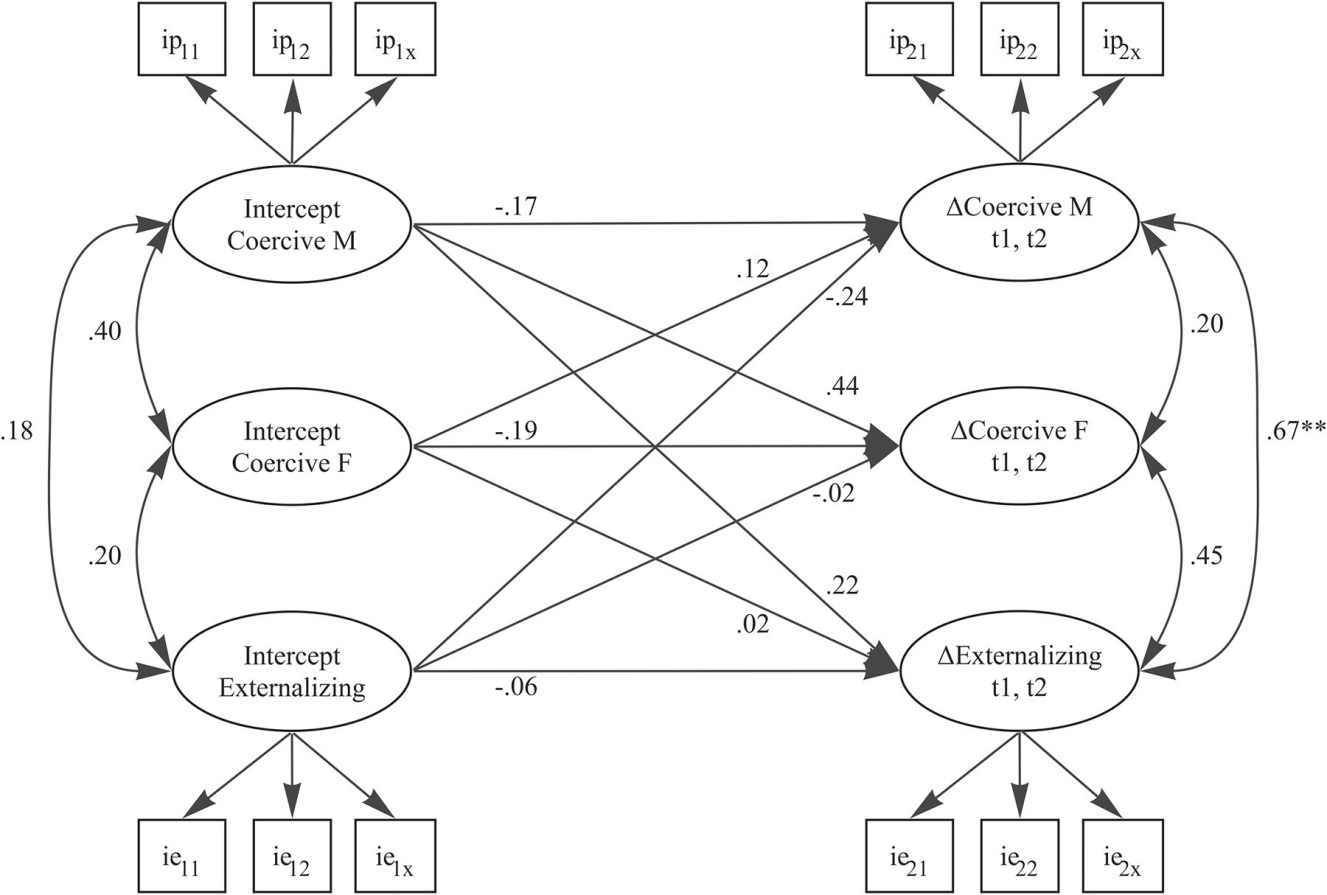
Interrelations between initial levels of, and changes in coercive parenting and child externalizing behavior. *Note*. Coefficients are standardized parameters. Similar to maternal coercive parenting and child externalizing behavior, a latent factor was created for paternal coercive parenting using observed indicators, but these are not shown for clarity of presentation. Residual variances of parallel items were allowed to correlate between T1 and T2. Coercive M = maternal coercive parenting. Coercive F = paternal coercive parenting. ip_11_ = parenting time 1, indicator 1; ie_11_ = externalizing behavior time 1, indicator 1, etc. * *p* < .10. ** *p* < .05. *** *p* < .001.

The final multivariate latent change score model (Model 1) had an adequate fit: normed (χ^2^/df) Satorra-Bentler-scaled χ^2^ = 1.20, CFI = 0.923, TLI = 0.917, RMSEA = 0.045, and SRMR = 0.090. Fig 1 depicts the interrelations between initial levels of, and changes in, maternal and paternal coercive parenting and child externalizing behavior. Table 3 summarizes the model parameters, covariances, and longitudinal coupling effects between initial levels of and change scores of coercive parenting and child externalizing behavior. On average, maternal coercive parenting, paternal coercive parenting, and child externalizing behavior did not change across the measurement occasions (see means of change scores), however there were within-person changes in mothers’ coercive parenting and children’s externalizing behavior over time (see variance of change scores). Initial levels of coercive parenting and child externalizing behavior were unrelated to changes in these variables (see longitudinal coupling effects). Changes in maternal coercive parenting were significantly and positively related to changes in child externalizing behavior (*B* = 0.02, *SE* = 0.01, *p* = .009). That is, when mothers increased in coercive parenting over time, higher levels of child externalizing behavior were reported. However, changes in paternal coercive parenting were not associated with changes in child externalizing behavior (*B* = 0.02, *SE* = 0.01, *p* = .098). Consistent with the finding that the magnitude and direction of the associations between changes in coercive parenting and child externalizing behavior were fairly the same across mothers and fathers, a Wald test indicated that these associations did not differ significantly between fathers and mothers (χ^2^(1) = 0.016, *p* = .898).

**Table 3:** Model parameters, covariances, and longitudinal coupling effects between intercepts and change scores in the final multivariate latent change score model.

						Intercepts				Change Scores					
						Coerc M_1_		Coerc F_1_		ΔCoerc M_1,2_		ΔCoerc F_1,2_		ΔExtern_1,2_	
		Means		Variance		Covariance				Longitudinal coupling effects					
		*B*	*SE*	*B*	*SE*	*B*	*SE*	*B*	*SE*	*B*	*SE*	*B*	*SE*	*B*	*SE*
**Intercepts**	**Coerc M**_**1**_	0^a^	-	0.06	0.04	-	-	-	-	-0.14	0.22	0.49	0.40	0.15	0.19
	**Coerc F**_**1**_	0^a^	-	0.10**	0.04	0.03	0.02	-	-	0.07	0.13	-0.16	0.20	0.01	0.11
	**Extern**_**1**_	0^a^	-	0.06**	0.02	0.02	0.01	0.02	0.01	-0.19	0.17	-0.02	0.29	-0.04	0.14
										Covariance					
										*B*	*SE*	*B*	*SE*	*B*	*SE*
**Change Scores**	**ΔCoerc M**_**1,2**_	0.05	0.03	0.03*	0.02	-	-	-	-			-	-	-	-
	**ΔCoerc F**_**1,2**_	0.09	0.05	0.06	0.04	-	-	-	-	0.01	0.01	0.02	0.01	-	-
	**ΔExtern**_**1,2**_	0.03	0.03	0.03**	0.01	-	-	-	-	0.02**	0.01	-	-	-	-

*Note*. Coerc M_1 =_ maternal coercive parenting at T1. Coerc F_1 =_ paternal coercive parenting at T1. Extern_1_ = child externalizing behavior at T1. ΔCoerc M_1,2_ = change in maternal coercive parenting across T1 and T2, etc. ^a^ We constrained intercept means to 0 for model identification purposes.

* *p* < .10.

** *p* < .05.

*** *p* < .001.

### Moderation by changes in the other parent’s coercive parenting

Based on Akaike Information Criterion (AIC) and Bayesian Information Criterion (BIC), model fit of Model 2 worsened with the addition of the interaction term between changes in maternal coercive parenting and changes in paternal coercive parenting (ΔAIC = 1.97, ΔBIC = 4.52). With regards to the exploratory Hypothesis 2, the relation between changes in one parent’s coercive parenting and changes in child externalizing behavior was not significantly moderated by changes in the other parent’s coercive parenting (*B* = -0.12, *SE* = 0.66, *p* = .861).

### Moderation by parent-child attachment relationship quality

Model fit of Model 3 worsened with the addition of the interaction term between changes in maternal coercive parenting and mother-child attachment relationship quality (ΔAIC = 1.74, ΔBIC = 4.30). In contrast to Hypothesis 3, mother-child attachment relationship did not moderate the relation between changes in maternal coercive parenting and changes in child externalizing behavior (*B* = -0.27, *SE* = 0.54, *p* = .610).

Model fit also worsened with the addition of the interaction term between changes in paternal coercive parenting and father-child attachment relationship quality (ΔAIC = 1.95, ΔBIC = 4.51). In contrast to Hypothesis 3, father-child attachment relationship quality did not moderate the relation between changes in paternal coercive parenting and changes in child externalizing behavior (*B* = -0.19, *SE* = 0.96, *p* = .842).

### Robustness checks

We conducted sensitivity analyses to examine the robustness of our results when we additionally controlled for the time difference between measurement occasions. The results of the final multivariate latent change score model with and without the covariate time difference between measurement occasions were consistent.

## Discussion

Embedded within the frameworks of social learning [20], coercive family processes [21, 22], family systems theory [12], and attachment theory [17, 40], we investigated whether and to what extent changes in mothers’ and fathers’ coercive parenting were related to changes in child externalizing behavior across the pre-pandemic period and the onset of the first COVID-19 lockdown in the Netherlands. Furthermore, we examined to what extent changes in the other parent’s coercive parenting, and the quality of mother-child and father-child attachment relationships moderated these linkages. Expanding upon previous works on interrelated changes in coercive parenting and child externalizing behavior [7, 14], we found that changes in mothers’, but not fathers’ coercive parenting were positively related to changes in child externalizing behavior. However, our data did not reveal that the level of change in coercive parenting of one parent shaped the association between changes in the other parent’s coercive parenting and changes in child externalizing problems. Moreover, neither mother-child nor father-child attachment relationship quality moderated the associations between changes in coercive parenting and changes in child externalizing behavior.

### Changes in coercive parenting and changes in child externalizing behavior

In the present study, we scrutinized the role of mothers and fathers in the associations between changes in coercive parenting and changes in child externalizing behavior. Although, on average, coercive parenting and child externalizing behavior did not change across the pre-pandemic period and the onset of the first COVID-19 lockdown, we found intraindividual interrelated changes in maternal, but not paternal, coercive parenting and child externalizing behavior. These findings suggest that, even though there was not one pattern of change on average, within-person changes in mothers’ coercive parenting and children’s externalizing behavior took place over time. However, the initial levels of coercive parenting and child externalizing behavior were unrelated to changes in parent and child behavior over time. These latter findings indicate that any changes in parental coercive parenting and child externalizing behavior were not explained by initial, pre-pandemic levels of parent and child behavior. Our main results revealed that changes in maternal, but not paternal, coercive parenting were positively related to changes in child externalizing behavior. Of note, the interrelated changes in coercive parenting and child externalizing behavior were consistent across mothers and fathers in that both associations were positive and of the same magnitude. The lack of significant difference in the interrelated changes in parent and child behavior across mothers and fathers also indicates that not finding a significant association between changes in fathers’ coercive parenting and child externalizing behavior may be due to low study power. Future studies with a larger sample size are needed to replicate our findings.

In general, our results resonate with findings from earlier studies [7, 14, 25]. Our results additionally show that for mothers, changes in their coercive parenting are associated with changes in child externalizing behavior over and above changes in the fathers’ coercive parenting. As such, our findings provide support for the transactional processes that underlie coercive, hostile, or aversive behaviors used by mothers and children in sudden stressful contexts, highlighting that within families, increases (or decreases) in coercive parenting are related to increases (or decreases) in child externalizing behavior over time. Our results also hint towards similar transactional processes between father and child behavior but call for replication by studies with more study power.

With regards to our exploratory hypothesis, we did not find a strengthening, interfering, or a buffering association of changes in one parent’s coercive parenting on changes of the other parent’s coercive parenting in relation to changes in child externalizing behavior. We can explain the lack of interaction by the lack of within-person variance found in the level of fathers’ coercive parenting across T1 and T2, suggesting that there was stability in coercive parenting within fathers. Moreover, within-person variance in changes in maternal coercive parenting and child externalizing behavior suggest heterogeneity in the pattern of changes in parent and child behavior, such that some individuals increased, while others decreased, in the respective behavior. It might have been the case that different patterns present in our data cancelled each other out, or that the number of families per pattern was too small to be able to detect a significant interaction.

### Parent-child attachment relationship as moderator

Our study showed that the quality of mother-child and father-child attachment relationships did not moderate the associations between changes in coercive parenting and changes in child externalizing behavior. These results are inconsistent with previous research examining moderation by mother-child or father-child attachment relationship quality or similar contexts, such as parental warmth in the associations between harsh parenting and child externalizing behavior [27, 28, 38, 61]. Three explanations may provide insight into why our results diverge from previous studies. First, in our convenience sample, there were only a few families in our data with relatively low levels of mother-child and father-child attachment relationship quality, with the consequence that we might have had too little variation to detect an interaction. Second, our study findings should be interpreted in light of the latent change scores that we used. While in previous studies secure parent-child attachment relationship quality moderated the relation between absolute levels of negative parenting and absolute levels of child behavior [27, 28], our findings shed light onto the role of parent-child attachment relationship in the association between changes in parenting and child behavior over time. Our findings suggest that the previously found protective role of parent-child attachment relationship quality in cross-sectional associations [27, 28] may not extend to associations in which interindividual (between-person) differences in intraindividual (within-person) changes are examined. Third, with regards to the family systems theory, a three-way interaction may take place between change in maternal coercive parenting, change in paternal coercive parenting, and parent-child attachment relationship quality, in relation to changes in child externalizing behavior. For example, it is possible that a strengthening pattern of interaction between changes in maternal and paternal coercive parenting in relation to child externalizing behavior only presents itself for children with a less secure attachment relationship with their parent(s). Given our limited sample size and study power, we were unable to test three-way (or even four-way) interactions. We recommend future studies with larger sample sized and more variation in parent-child attachment relationship quality to employ a repeated measures design to replicate and untangle the extent to which parent-child attachment relationship could moderate the (moderated) interrelated changes in coercive parenting and child externalizing behavior.

### Strengths, limitations, and future work directions

Several aspects of the current study are considerable strengths. Despite the theoretical underpinnings of the transactional and interrelated changes in parent and child behavior, previous studies often did not statistically test changes over time, but instead included mean level scores of parent and child behavior. Shortcomings of studies that did investigate changes in parent and child behavior over time in the context of COVID-19 included relying on retrospective assessment of parent and child behavior, which were potentially influenced by recall bias [4, 13]. By including data collected prior to the pandemic and at the onset of the lockdown, we were able to employ advanced methods to study the interrelation between interindividual (between-person) differences in intraindividual (within-person) changes in coercive parenting and child externalizing behavior. Furthermore, there was low attrition across the two measurement occasions, and missings were likely occurring completely at random. This is advantageous given our modest sample size and the fact that studies conducted during the COVID-19 pandemic may be especially prone to non-response bias, which may lead to threats of sample validity [62]. Moreover, by taking a family systems perspective, our study includes information on both mothers and fathers from the same family, and thereby contributes to the growing literature on the role of multiple caregivers in child development. Specifically, we incorporated both mothers’ and fathers’ self-reports on coercive parenting and reports on child externalizing behavior, as well as observer-rated mother-child and father-child attachment relationship quality using the AQS [53]. The use of reliable and validated measurements from multiple informants limited the potential for single source bias.

Still, our findings should be considered in light of some limitations. First, while our findings provide support for the well-grounded theories on social learning and coercive family processes, we cannot untangle the directionality of the linkages between changes in parents’ coercive parenting and child externalizing behavior. With our covariance analyses between change scores, however, we did not impose directionality on the potential bidirectional relationship between parent and child behavior.

Second, despite having a prospective study design in the context of the COVID-19 pandemic, the current study was limited to data from two measurement occasions, which restricted us from examining the pattern of interrelated changes in parent and child behavior after lockdown measures were uplifted and later again tightened. Future studies could include additional data collection waves, especially with regards to the COVID-19 context, and examine both between-family and within-family associations as well as parent-driven and child-driven effects in order to gain a better insight into dynamic coercive family processes in the context of changing environmental stressors.

Third, having data collected prior to the pandemic and during the onset of the first COVID-19 lockdown in the Netherlands provided us with a natural experiment without a control group, in which the pandemic may be regarded as exposure to stressors that are outside of the researchers’ control. Without a control group, however, we are unable to deduce whether any changes in coercive parenting and child externalizing behavior were due to the pandemic or related regulations, or other potentially stressful life events, such as children’s transition to primary school, changes in family structure, parents’ jobs, or parents’ mental health.

Fourth, we excluded an original item from the coercive parenting subscale of the PAFAS. We recommend any comparisons between our results and those in other studies [2] to be made in light of the adjusted coercive parenting subscale in the present study, which does not include any items tapping into physical coercive behavior. Future studies are needed to replicate the longitudinal measurement invariance in the coercive parenting subscale of the PAFAS for mothers and fathers.

Finally, although we did not aim to recruit a clinical sample, the generalizability of our findings is limited to families who were relatively well off during the onset of the lockdown, as described in Lucassen et al. [2] on the same dataset as the present study. It is plausible that the interrelated changes in coercive parenting and child externalizing behavior are stronger in families dealing with multiple co-occurring contextual and interpersonal stressors [63, 64], and that our conclusion should therefore be seen as conservative.

### Implications for practice

Our findings on the interrelated changes in coercive parenting and child externalizing behavior across the pre-pandemic period and the onset of the first COVID-19 lockdown have important implications for clinical practice. As coercive parent-child interactions, through negative reinforcement contingencies [23], may increase during lockdowns and non-normative stressful events, support should be provided to both parents and children to reduce stress-related coercive interactions by adapting the reinforcement of coercive behavior [65]. Given that parents frequently reported having positive outlooks besides negative emotions and moods during COVID-19 [66], it may be particularly important to help parents and children foster positive outlooks, which may, in turn, help in adapting the reinforcement of coercive interaction patterns. Therefore, we recommend that preventions strategies and recovery tools used during and after future lockdowns or non-normative stressful events [67] include evidence-based (parenting) support to both mothers, fathers, and young children.

## Conclusions

Within the frameworks of social learning, family systems theory, and attachment theory, the present study expanded upon previous research to examine the interrelated changes in coercive parenting and child externalizing behavior across the pre-pandemic period and the onset of the first COVID-19 lockdown in the Netherlands, and the moderating roles of changes in the other parent’s coercive parenting and parent-child attachment relationship quality. We found that changes in mothers’, but not fathers’ coercive parenting were independently related to changes in child externalizing behavior. These associations were moderated by neither changes in the other parent’s coercive parenting, nor by the quality of mother-child and father-child attachment relationship.

## Supporting information

S1 File Supporting information. Supplementary material including Figures S1 and S2, Measurement Invariance Tests, Tables S1 and S2.
(PDF)
