# Supplementary material for: Changes in coercive parenting and child externalizing behavior across COVID-19 and the moderating role of parent-child attachment relationship quality
Source: PLoS One. 2023 Oct 12;18(10):e0290089. doi: 10.1371/journal.pone.0290089 (PMC10569510; doi:10.1371/journal.pone.0290089)
Supplement: S1 File — Supplementary material including Figures S1 and S2, Measurement Invariance Tests, Tables S1 and S2. (PDF) [file pone.0290089.s001.pdf]

Changes in coercive parenting and child externalizing behavior across COVID-19 and the moderating role of parent-child attachment relationship quality

Supplementary Material

Figure S1.

Latent variables maternal coercive parenting and paternal coercive parenting.

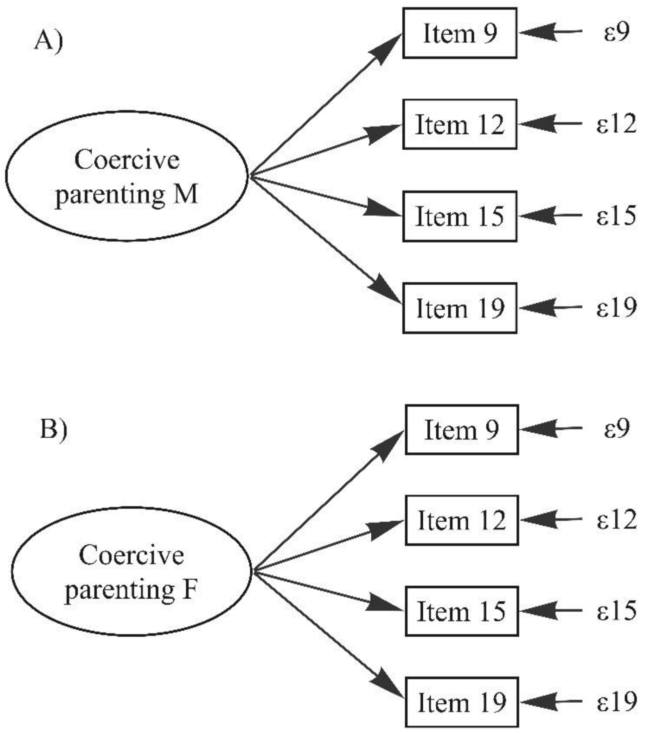

Note. Coercive parenting M = maternal coercive parenting. Coercive parenting F = paternal coercive parenting. Figure depicts the selected original items from the coercive parenting subscale (Sanders et al., 2014) that load onto the latent construct maternal coercive parenting (A) and paternal coercive parenting (B) at both measurement occasions.

Figure S2.

Latent variable child externalizing behavior

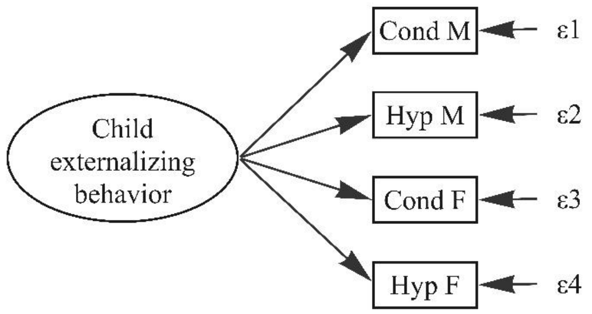

Note. Cond M = mother-reported conduct problems scale, Cond F = father-reported conduct problems scale, Hyp M = mother-reported Hyperactivity/Inattention scale, Hyp F = father-reported Hyperactivity/Inattention scale. Figure depicts the created parcels, Conduct problems (mother and father report) and Hyperactivity/Inattention (mother and father report) as observed indicators of the latent construct child externalizing behavior.

Measurement Invariance Tests

We present model fit indices and model comparisons in Tables S1 and S2. The initial models for coercive parenting showed configural noninvariance, as an original item ("I spank (smack) my child when they misbehave") did not load significantly for either parent at T2 (Model A_{mother}: $B = 0.11$, $SE = 0.10$, $p = .289$; Model A_{father}: $B = 0.15$, $SE = 0.10$, $p = .116$). This item is the only item tapping into physical coercive parenting in PAFAS, therefore, we excluded it from the measurement models for maternal and paternal coercive parenting. Future studies are needed to replicate longitudinal measurement invariance in the mother and father-reported coercive parenting subscale of the PAFAS. For paternal coercive parenting, we added a residual error covariance between two items at T2. Further, we found configural, metric, scalar, and strict invariance across the two measurement occasions for maternal and paternal coercive parenting. The final measurement models for maternal and paternal coercive parenting had good model fit (see Table S2), and the unstandardized factor loadings ranged between .72 and 1.39 for mothers and between .51 and 1.10 for fathers.

For the measurement models of child externalizing behavior assessing invariance across reporter at T1 (see Table S1), we added an error covariance between two indicators for mothers, and at T2, we added an error covariance between a mother-reported and a father-reported indicator. For the measurement models assessing longitudinal measurement invariance (see Table S2), we added error covariances between six indicators. Further, we found configural, metric, scalar, and strict invariance across reporters and measurement occasions for child externalizing behavior. The final measurement model for child externalizing behavior had a good fit (Table S2), and the unstandardized factor loadings ranged between 1.00 and 1.39.

Table S1.

Model Fit Indices and Model Comparisons of Measurement Models: Assessing Invariance Across Reporter (Mother versus Father)

	Model Fit Indices								Model comparison						
				Scaling							Δ				
Construct	Model	χ^2SB	df	factor	CFI	TLI	RMSEA	SRMR	Comparison	ΔCFI	RMSEA	ΔSRMR	Δ χ^2SB	df	*p*
Externalizing	A[1]	1.192	1	1.5653	0.997	0.982	0.045	0.021							
behavior T1	B	3.784	3	1.0107	0.988	0.976	0.052	0.047	B vs A	-0.009	0.007	0.026	2.6706	2	0.263
	C	3.954	5	0.9923	1.000	1.000	0.000	0.049	C vs B	0.012	-0.052	0.002	0.1027	2	0.950
	**D**	**6.873**	**7**	**0.9980**	**1.000**	**1.000**	**0.000**	**0.059**	**D vs C**	**0.000**	**0.000**	**0.010**	**2.9002**	**2**	**0.235**
Externalizing	A[2]	1.272	1	1.3436	0.998	0.985	0.054	0.023							
behavior T2	B	3.368	3	0.9466	0.997	0.993	0.036	0.043	B vs A	-0.001	-0.018	0.020	1.9771	2	0.372
	C	3.440	5	0.9353	1.000	1.000	0.000	0.044	C vs B	0.003	-0.036	0.001	0.0319	2	0.984
	**D**	**7.643**	**7**	**0.8768**	**0.994**	**0.995**	**0.031**	**0.044**	**D vs C**	**-0.006**	**0.031**	**0.000**	**4.7689**	**2**	**0.092**

Note. Model fit indices in bold indicate chosen model. Model A = configural model. Model B = metric model. Model C = scalar model. Model D = strict model. Adjustments: [1] Error covariance between two indicators (Hyperactive/Inattentive and Conduct problems parcels) added for mothers. [2] Error covariance between a mother-reported (Conduct problems parcel) and a father-reported indicator (Hyperactive/Inattentive parcel) added.

Table S2.

Model Fit Indices and Model Comparisons of Measurement Models: Assessing Invariance Across Measurement Occasions (T1 versus T2)

	Model Fit Indices								Model comparison						
Construct	Model	χ^2SB	df	Scaling factor	CFI	TLI	RMSEA	SRMR	Comparison	ΔCFI	Δ RMSEA	Δ SRMR	Δ χ^2SB	df	*p*
Maternal	A	15.555	15	0.8390	0.996	0.993	0.020	0.037							
coercive	B	21.789	18	0.8377	0.976	0.962	0.047	0.053	B vs A	-0.020	0.027	0.016	6.2584	3	0.100
parenting	C	25.283	21	0.8608	0.972	0.963	0.046	0.060	C vs B	-0.004	-0.001	0.007	3.5131	3	0.319
	**D**	**30.038**	**25**	**0.9338**	**0.968**	**0.964**	**0.046**	**0.068**	**D vs C**	**-0.004**	**0.000**	**0.008**	**4.7727**	**4**	**0.311**
Paternal	A[1]	14.699	14	1.0633	0.994	0.989	0.023	0.050							
coercive	B	17.844	17	1.1361	0.993	0.989	0.023	0.061	B vs A	-0.001	0.000	0.011	3.1461	3	0.370
behavior	C	21.262	20	1.1007	0.990	0.986	0.026	0.067	C vs B	-0.003	0.003	0.006	3.4780	3	0.324
	**D**	**23.594**	**24**	**1.0740**	**1.000**	**1.000**	**0.000**	**0.073**	**D vs C**	**0.010**	**-0.026**	**0.006**	**2.0594**	**4**	**0.725**
Externalizing	A[2]	25.494	24	0.9854	0.995	0.994	0.026	0.067							
behavior	B	25.431	25	0.9913	0.998	0.998	0.013	0.067	B vs A	0.003	0.013	0.000	0.0776	1	0.781
	C[3]	24.931	24	0.9939	1.000	1.000	0.000	0.068	C vs B	0.002	-0.013	0.001	-	0	-
	**D**	**29.291**	**27**	**0.9917**	**0.992**	**0.991**	**0.030**	**0.073**	**D vs C**	**-0.008**	**0.030**	**0.005**	**4.3825**	**3**	**0.223**

Note. Model A = configural model. Model B = metric model. Model C = scalar model. Model D = strict model. Adjustments: [1] Error covariance between two indicators (original items #12 and #15, Sanders et al., 2014) added at T2. [2] Error covariance between two father-reported indicators (Conduct problems and Hyperactive/Inattentive

parcels) added at T1. [3] Error covariance between a mother-reported indicator (Hyperactive/Inattentive parcel) at T2 and a father-reported indicator (Hyperactive/Inattentive parcel) at T1 added.

Reference

Sanders, M. R., Morawska, A., Haslam, D. M., Filus, A., & Fletcher, R. (2014). Parenting and Family Adjustment Scales (PAFAS): Validation of a brief parent-report measure for use in assessment of parenting skills and family relationships. *Child Psychiatry and Human Development*, *45*(3), 255–272. https://doi.org/10.1007/s10578-013-0397-3
